# Possible Capecitabine-Induced Coronary Vasospasm and Myocardial Infarction

**DOI:** 10.7759/cureus.81890

**Published:** 2025-04-08

**Authors:** Deepa Soodi, Vinod Kumar Reddy Cirra, Guruprasad D Naik, Param Sharma

**Affiliations:** 1 Cardiology, Marshfield Clinic Health System, Marshfield, USA; 2 Internal Medicine, Deccan College of Medical Sciences, Hyderabad, IND; 3 Cardiology, Goa Medical College, Bambolim, IND

**Keywords:** capecitabine side effects, chemotherapy-related toxicity, coronary artery vasospasm, myocardial infarction, st-segment elevation myocardial infarction (stemi)

## Abstract

Capecitabine is a 5-fluorouracil precursor, approved for breast and colorectal cancer. We report a case of capecitabine-induced coronary vasospasm. A 77-year-old woman with breast cancer receiving capecitabine presented with chest pain, and an electrocardiogram demonstrated acute anterior-lateral myocardial infarction. Coronary angiography demonstrated normal coronary arteries. Discontinuation of capecitabine resolved symptoms. Failing to identify the cardiotoxicity associated with capecitabine could have had serious consequences.

## Introduction

Coronary vasospasm is an uncommon but important nonatherosclerotic cause of acute coronary syndromes. Previous studies have documented that drugs such as ephedrine-based sympathomimetic drugs, sumatriptan, and 5-fluorouracil (FU) have been documented to cause coronary vasospasm and related acute coronary syndromes [1]. In more recent years, capecitabine, a 5-FU precursor, has been identified as a cause for coronary vasospasm. We present a patient with chest pain and ST elevation by electrocardiogram (ECG) who had initiated capecitabine three days before this presentation. Atherosclerotic coronary artery disease was excluded. Dynamic ECG changes confirmed coronary vasospasm relieved with sublingual/IV and oral nitrates. Discontinuation of capecitabine led to the cessation of any further symptoms.

## Case presentation

A 77-year-old woman with a history of hypertension and paroxysmal atrial fibrillation presented with severe chest pain described as “raw feeling” in the chest. The chest pain was associated with shortness of breath, waxing, and waning without complete relief for over 10 hours. The pain was nonpleuritic and not relieved with antacid.

The patient's past medical history was notable for hypertension, paroxysmal atrial fibrillation, anticoagulation using apixaban, osteopenia, and breast cancer. Six months earlier, the patient had been diagnosed with invasive ductal carcinoma of the right breast (triple negative; stage cT1c N0 M0, Stage IB). She received four cycles of neoadjuvant docetaxel and cyclophosphamide, followed by right breast lumpectomy and sentinel node biopsy. The pathologic stage was T1cN0. A heterozygous BRCA1 mutation of uncertain significance was present. Capecitabine was initiated at a dose of 1,500 mg twice daily.

**Figure 1:**
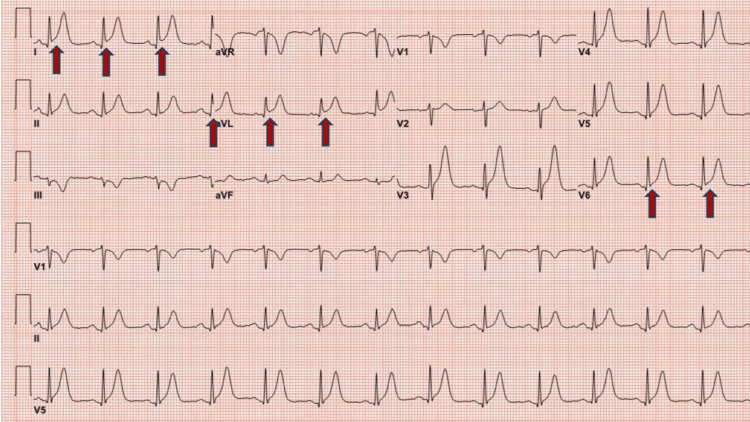
ECG showing ST elevation in multiple leads (red arrows) ECG: electrocardiogram; aVR: augmented vector right; aVL: augmented unipolar lead; aVF: augmented vector foot

**Figure 2:**
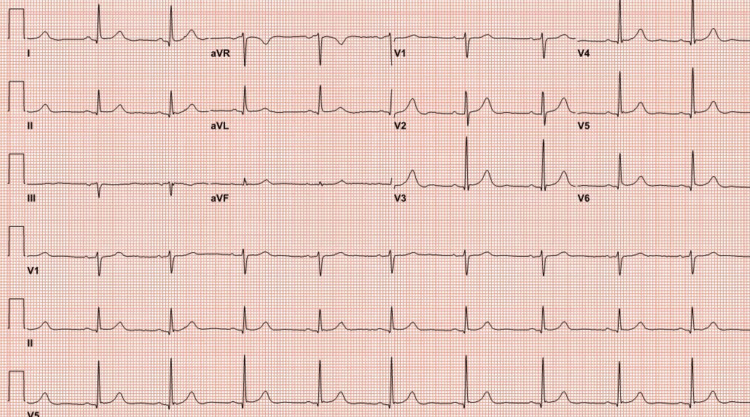
ECG with resolution of ST elevation ECG: electrocardiogram; aVR: augmented vector right; aVL: augmented unipolar lead; aVF: augmented vector foot

**Figure 3:**
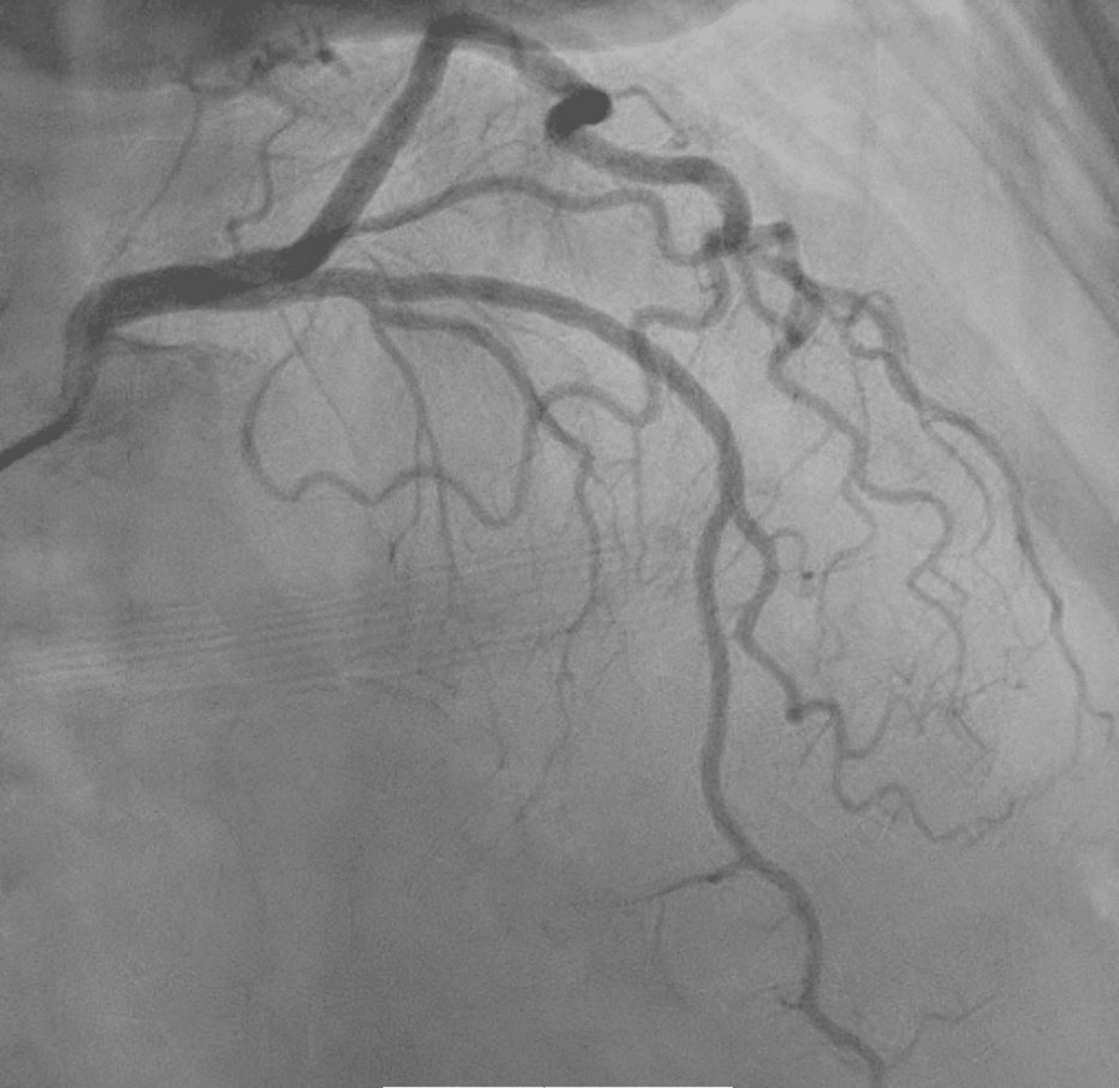
Coronary angiogram showing normal left coronary arteries

**Figure 4:**
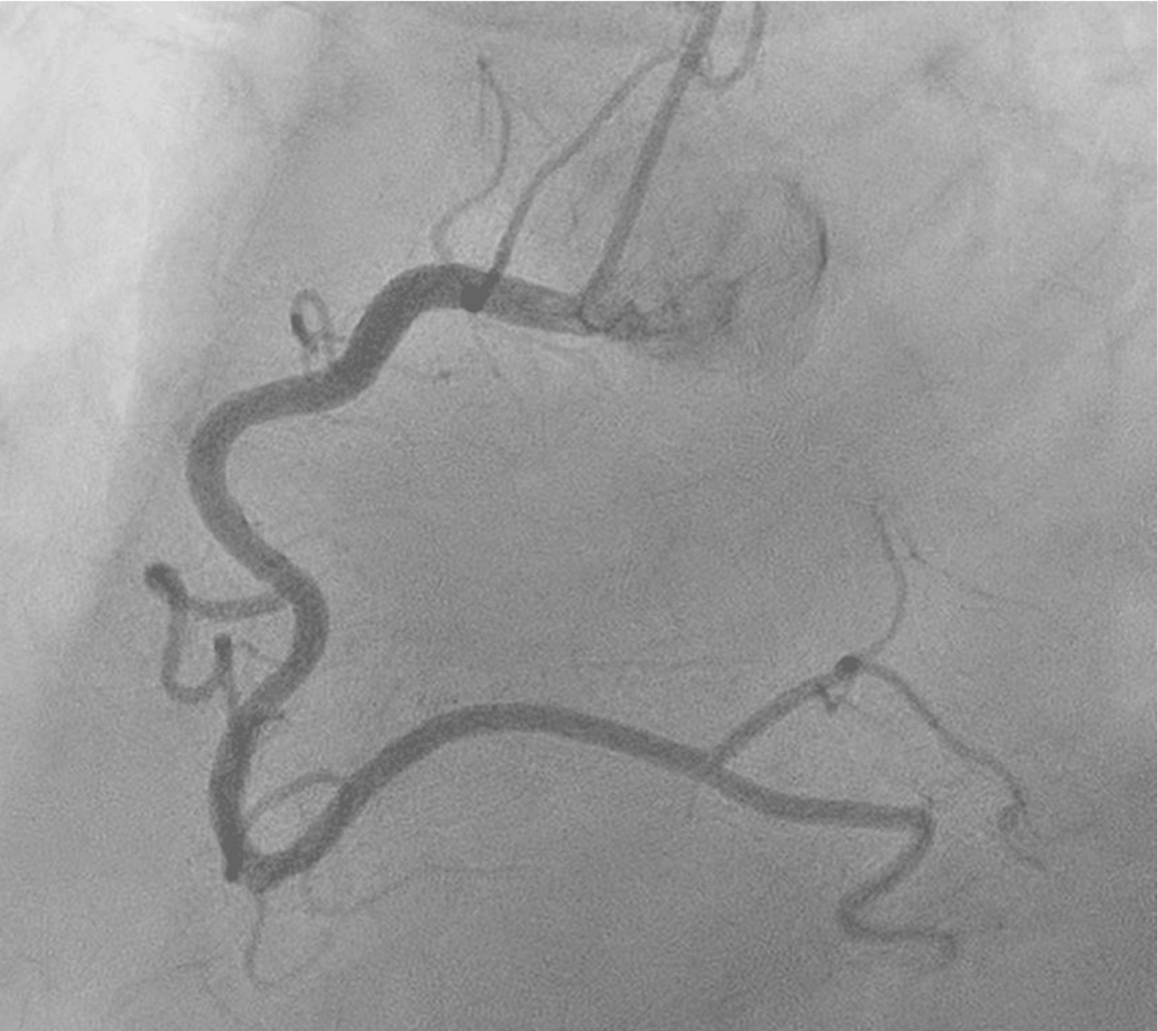
Coronary angiogram showing normal right coronary artery

**Figure 5:**
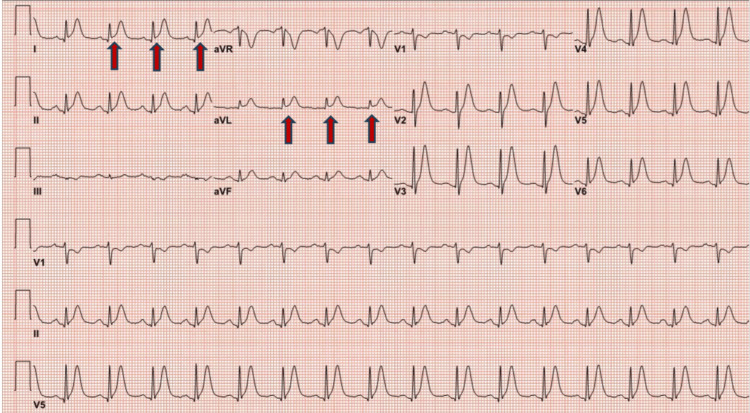
ECG with ST elevations in multiple leads (red arrows) ECG: electrocardiogram; aVR: augmented vector right; aVL: augmented unipolar lead; aVF: augmented vector foot

The patient developed chest pain after 48 hours of the first dose of capecitabine. The ECG at presentation showed ST elevation in lead l, augmented limb lead aVF, and augmented precordial lead V6 along with tall T waves suggestive of acute myocardial infarction (Figure 1). Initial troponin I was normal, and chest pain was relieved with sublingual nitroglycerine, with repeat ECG indicating resolution of ST elevation (Figure 2). The patient underwent early coronary angiography given the transient ST elevation that showed normal coronary arteries (Figure 3 shows the left coronary arteries, and Figure 4 shows the right coronary artery). Two hours later, the patient developed recurrent severe chest pain again, accompanied by ST elevation in multiple leads (Figure 5).

The patient was then treated with IV nitroglycerine, which was transitioned to oral nitrates along with oral diltiazem. Subsequent troponin I was elevated while the echocardiogram showed a normal left ventricular ejection fraction and no wall motion abnormality. Table 1 shows the patient's normal lab values. The patient was discharged home a day later after being asymptomatic for over 24 hours on oral nitrates and oral diltiazem. Capecitabine was discontinued. At one-month follow-up, the patient remained asymptomatic with no recurrent chest pain symptoms. The 24-hour Holter monitor did not reveal any transient ST-segment depressions or elevations.

**Table 1:** Patient lab values and normal reference lab values

Variables	Patient lab values	Normal lab values
High-sensitivity troponin: 0 hours	14 ng/L	0-57 ng/L
High-sensitivity troponin: 2 hours	181 ng/L	0-57 ng/L

## Discussion

Capecitabine cardiotoxicity depends on various factors such as dose, cardiac comorbidities, and schedule of chemotherapy [1]. The proposed mechanism of cardiotoxicity includes endothelial-dependent and endothelial-independent mechanisms. Endothelial dysfunction causes impaired vessel lumen regulation, resulting in decreased nitric oxide (NO) and increased endothelin, which promotes vasoconstriction and coronary vasospasm. During endothelial-independent mechanisms, smooth muscle dysfunction leads to vasoconstriction with preserved endothelial NO release [2]. Prior ischemic heart disease strongly predicts capecitabine-induced cardiotoxicity [3]. However, there are several cases reported of capecitabine cardiotoxicity in patients without prior cardiovascular disease [4]. Patients experiencing symptoms similar to variant angina present with chest pain at rest, and it is possible that they may or may not exhibit ECG changes indicative of ischemia. The use of capecitabine has been associated with exercise-induced ECG changes [5]. Goldsmith et al. [4] reported a case of capecitabine cardiotoxicity, manifested as exercise-induced global myocardial ischemia with normal coronary arteries and left ventricular function.

Capecitabine therapy has been associated with the onset of chest pain, as observed in studies by Goldsmith et al. [4] and Wijesinghe et al. [6] who reported an acute coronary syndrome developed in a patient after two days of capecitabine treatment. Additionally, Goldsmith et al. [4] documented symptoms of coronary vasospasm a few days following the initiation of capecitabine therapy. In our case, the patient presented with chest pain two days after starting capecitabine. It is crucial to remember that, depending on the dosage, cardiac side effects can appear within 24 hours after taking the medicine [7].

Diagnostic testing, including ECG, may show ST-segment elevation, echocardiogram findings of regional wall motion abnormalities, and even segmental dyskinesis during the episodes of chest pain [8]. A coronary angiogram may reveal nonobstructive coronary arteries.

The primary treatment modality is the discontinuation of the drug. Lower dose capecitabine rechallenging has been studied in a retrospective study of 668 patients [9]. A prospective study of 664 patients [10] reported benefits from dose reduction and the activation of antianginal drugs at retreatment and with strict cardiac monitoring. According to a retrospective study by Jensen and Sørensen [9], nitroglycerin relieved symptoms. Prophylactic calcium channel blockers failed to show any effect on cardiac toxicity [10]. However, our patient was switched to olaparib for breast cancer treatment.

## Conclusions

In summary, our patient had capecitabine-induced coronary vasospasm in the absence of preexisting coronary artery disease. Coronary vasospasm can be reversed with the stopping of capecitabine. Failing to identify the cardiotoxicity associated with capecitabine could have had serious consequences. This case reports describe a single patient's experience, which may not be representative of all patients taking capecitabine. More extensive studies are needed to determine the true incidence and risk factors for capecitabine-induced coronary vasospasm. considering the seriousness of this effect.
